# Buruli Ulcer Lesions in HIV-Positive Patient

**DOI:** 10.3201/eid1604.091343

**Published:** 2010-04

**Authors:** Kapay Kibadi, Robert Colebunders, Jean-Jacques Muyembe-Tamfum, Wayne M. Meyers, Françoise Portaels

**Affiliations:** Institute of Tropical Medicine, Antwerp, Belgium (K. Kibadi, R. Colebunders, F. Portaels); Institut National de Recherche Biomédicale, Kinshasa, Democratic Republic of Congo (K. Kibadi, J.-J. Muyembe-Tamfum); University of Kinshasa, Kinshasa (K. Kibadi, J.-J. Muyembe-Tamfum); University of Antwerp, Antwerp (R. Colebunders); Armed Forces Institute of Pathology, Washington, DC, USA (W.M. Meyers)

**Keywords:** Buruli ulcer, HIV, rifampin, streptomycin, bacteria, letter

**To the Editor:** *Mycobacterium ulcerans* disease (Buruli ulcer) is a neglected and emerging tropical disease (*1*). It often leads to extensive destruction of skin and soft tissue with the formation of large ulcers (*2*). In 2004, the World Health Organization (WHO) recommended the combination treatment of rifampin/streptomycin for patients with this disease (*3*). According to WHO, development of new antimicrobial drug treatment is one of the major advances since the establishment of the Global Buruli Ulcer Initiative (*1*). Treatment with rifampin/streptomycin for >4 weeks can inhibit the growth of *M*. *ulcerans* in preulcerative lesions (*4*). In other patients, despite 4 weeks of treatment, lesions may deteriorate. Whether this treatment is less efficacious in persons with HIV infection is unknown.

In August 2008, a 35-year-old man was referred to the Medical Centre of the Democratic Republic of Congo for assessment of chronic ulcers. Lesions had appeared 12 months earlier when the patient was living in Kafufu/Luremo, a new focus of Buruli ulcer in Angola (*5*). Tissue specimens were subjected to Ziehl-Neelsen staining, culture, and PCR. All results were positive for *M*. *ulcerans*. Histopathologic analysis of formalin-fixed tissue confirmed the diagnosis of active Buruli ulcer. We treated the patient with a combination of rifampin (10 mg/kg/day, orally) and streptomycin (15 mg/kg/day, by intramuscular injection). Wound dressings containing an aqueous solution of chloramine/metronidazole/nitrofurantoine were changed daily (*6*). For logistic reasons, surgery (large excision) under general anesthesia was not possible.

Characteristics of the patient are shown in the Technical Appendix. At the start of treatment, the patient had a large ulcer on the right leg and thigh, a nodule 2 cm in diameter on the left thigh, and a plaque 8 cm in diameter on the left thigh. After 2 weeks of treatment, the size of the large ulcer had increased. After 4 weeks, the nodule became an ulcer 6 cm in diameter, and the plaque became a large ulcer 15 cm in diameter with a satellite ulcer 2 cm in diameter. After 8 weeks, we observed enlargement of all lesions and the appearance of an ulcer on the left wrist. Treatment was continued for an additional 4 weeks (total 12 weeks). Radiologic investigation did not disclose any bone destruction.

The patient was positive for HIV by the Determine HIV-1/2 test (Abbott Laboratories, Dainabot Co. Ltd., Tokyo, Japan), the Uni-Gold HIV test (Trinity Biotech PLC, Bray, Ireland), and the Genie II HIV-1/HIV-2 test (Bio-Rad, Marnes-la-Coquette, France). Results of PCR for *M*. *ulcerans* and Ziehl-Neelsen staining were positive for all specimens obtained during the 8 weeks of initial treatment. The patient died 2 weeks after treatment ended, just when antiretroviral treatment had been scheduled to begin.

Although the patient did not respond clinically to treatment with rifampin/streptomycin, whether the treatment also failed microbiologically is more difficult to prove. Results of PCRs performed during treatment remained positive. However, PCR does not differentiate between living and dead *M*. *ulcerans* bacteria. Therefore, our positive results suggest, but do not prove, treatment failure. The positive culture after 2 weeks of treatment also suggests treatment failure but cultures obtained at 4 and 8 weeks were contaminated. However, culturing *M*. *ulcerans* bacteria is difficult, especially if samples must be transported (*7*).

Patients with Buruli ulcer may also be infected with HIV. In a study conducted during January 2002–August 2003 that compared HIV prevalence in 426 patients with Buruli ulcer and 613 controls in southern Benin, HIV prevalence among patients with Buruli ulcer was higher (2.6%, 11/426) than among controls (0.3%, 2/613) (odds ratio 8.1) (*8*). However, none of these reported HIV-positive patients with Buruli ulcer were treated with rifampin/streptomycin and antiretroviral therapy (*8*).

A study of 224 patients with Buruli ulcer in Benin that evaluated the WHO-recommended regimen of 8 weeks of treatment with rifampin/streptomycin showed promising results (*9*). Chemotherapy alone was successful in achieving a cure rate of 47% of patients and was effective against ulcers <5 cm in diameter (*9*). However, HIV testing was not performed in this study. In Spain, an HIV-positive patient with aggressive, multifocal Buruli ulcer and osteomyelitis was cured by surgery, broad-spectrum antimicrobial drugs (not rifampin/streptomycin), and antiretroviral drugs (*10*). Relapse was not reported in this study at 6-months follow-up.

For control of Buruli ulcer in HIV-positive patients, patients should be treated with rifampin/streptomycin and antiretroviral therapy to stimulate their immunity. Our report emphasizes the urgent need to evaluate treatment of HIV-positive patients infected with Buruli ulcer with rifampin/streptomycin and antiretroviral drugs.

## Supplementary Material

Technical Appendix Characteristics of HIV-positive patient with Buruli ulcer during treatment with rifampin/streptomycin, Democratic Republic of Congo.

## References

[1] World Health Organization. Buruli ulcer: progress report. Wkly Epidemiol Rec. 2008;83:145–56. 18437758

[2] Portaels F, Silva MT, Meyers WM. Buruli ulcer. Clin Dermatol. 2009;27:291–305. 10.1016/j.clindermatol.2008.09.021 19362692

[3] World Health Organization. Provisional guidance on the role of specific antibiotics in the management of *Mycobacterium ulcerans* disease (Buruli ulcer) WHO/CDS/CPE/GBUI.10; 2004 [cited 2009 Dec 23]. http://www.who.int/buruli/information/antibiotics/en/index.html

[4] Etuaful S, Carbonnelle B, Grosset J, Lucas S, Horsfield C, Phillips R, Efficacy of the combination rifampin-streptomycin in preventing growth of *Mycobacterium ulcerans* in early lesions of Buruli ulcer in humans. Antimicrob Agents Chemother. 2005;49:3182–6. 10.1128/AAC.49.8.3182-3186.2005 16048922 PMC1196249

[5] Kibadi K, Panda M, Tamfum JJ, Fraga AG, Longatto Filho A, Anyo G, New foci of Buruli ulcer, Angola and Democratic Republic of Congo. Emerg Infect Dis. 2008;14:1790–2. 10.3201/eid1411.071649 18976574 PMC2630729

[6] Kibadi K. *Mycobacterium ulcerans* disease (Buruli ulcer): surgical treatment of 102 cases in the Democratic Republic of Congo [in French]. Med Trop (Mars). 2005;65:444–8. 16465813

[7] Eddyani M, Debacker M, Martin A, Aguiar J, Johnson CR, Uwizeye C, Primary culture of *Mycobacterium ulcerans* from human tissue specimens after storage in semi-solid transport medium. J Clin Microbiol. 2008;46:69–72. 10.1128/JCM.00301-07 17989199 PMC2224267

[8] Johnson RC, Nackers F, Glynn JR, de Biurrun Bakedano E, Zinsou C, Aguiar J, Association of HIV infection and *Mycobacterium ulcerans* disease in Benin. AIDS. 2008;22:901–3. 10.1097/QAD.0b013e3282f7690a 18427211

[9] Chauty A, Ardant MF, Adeye A, Euverte H, Guédénon A, Johnson C, Promising clinical efficacy of streptomycin-rifampin combination for treatment of Buruli ulcer (*Mycobacterium ulcerans* disease). Antimicrob Agents Chemother. 2007;51:4029–35. 10.1128/AAC.00175-07 17526760 PMC2151409

[10] Toll A, Gallardo F, Ferran M, Gilaberte M, Iglesias M, Gimeno JL, Aggressive multifocal Buruli ulcer with associated osteomyelitis in an HIV-positive patient. Clin Exp Dermatol. 2005;30:649–51. 10.1111/j.1365-2230.2005.01892.x 16197379

