# Supplementary material for: Buruli Ulcer Lesions in HIV-Positive Patient
Source: Emerg Infect Dis. 2010 Apr;16(4):738–9. doi: 10.3201/eid1604.091343 (PMC3321952; doi:10.3201/eid1604.091343)
Supplement: Technical Appendix — Characteristics of HIV-positive patient with Buruli ulcer during treatment with rifampin/streptomycin, Democratic Republic of Congo. [file 09-1343-Techapp.pdf]

Buruli Ulcer Lesions in HIV-Positive Patient

Technical Appendix

Characteristics of HIV-positive patient with Buruli ulcer during treatment with rifampin/streptomycin, Democratic Republic of Congo*

Period of treatment	Lesions	Clinical features	Results of microbiologic and histologic analyses
Day 1 (start of treatment)	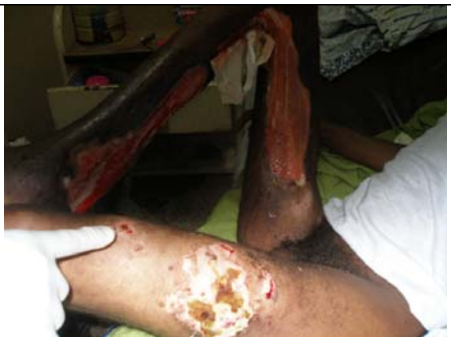	Large ulcer (right leg and thigh), nodule 2 cm in diameter (left thigh), and plaque 8 cm in diameter (left thigh)	ZN staining (ATS) (*1*) 2+, PCR positive, culture contaminated, active Buruli ulcer lesions
2 weeks	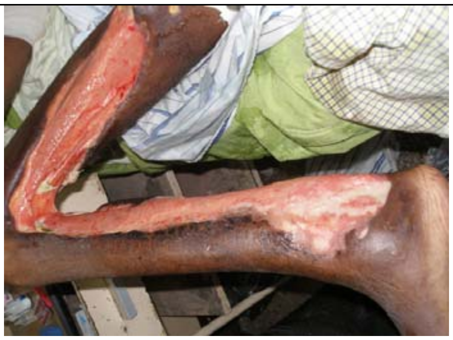	Increase in size of large ulcer (right leg and thigh)	ZN staining (ATS) 4+, PCR positive, culture positive, ND
4 weeks	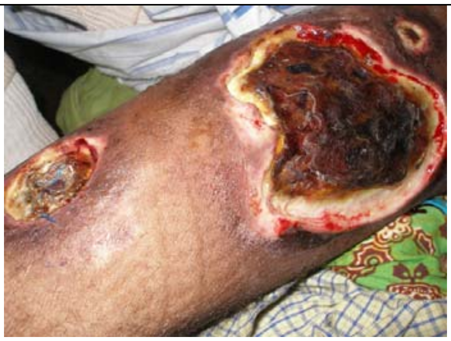	Nodule 2 cm in diameter (left thigh) becomes an ulcer 6 cm in diameter, plaque 8 cm in diameter (left thigh) becomes a large ulcer 15 cm in diameter, and appearance of an ulcer 2 cm in diameter	ZN (ATS) 1+, PCR positive, culture contaminated, ND

8 weeks	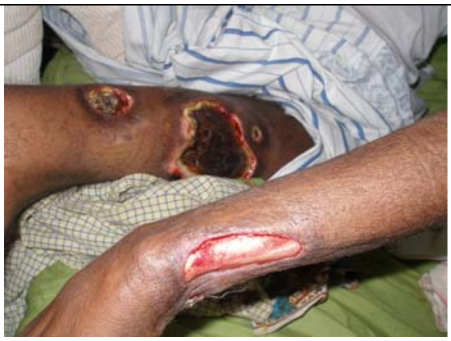	Increase in size of all lesions and appearance of an ulcer on the left wrist	ZN (ATS) 4+, PCR positive, culture contaminated, ND

*ZN, Ziehl-Neelsen; ATS, American Thoracic Society; ND, histologic analysis not done.

Reference

1. American Thoracic Society. Diagnostic standards and classification of tuberculosis and other mycobacterial diseases (14th edition). Am Rev Respir Dis. 1981;123:343–58. PubMed
